# Who accessed STI testing in Britain during the COVID-19 pandemic and how: Findings from Natsal-COVID, a cross-sectional quasi-representative survey

**DOI:** 10.1177/09564624241277582

**Published:** 2024-09-11

**Authors:** Nuria Gallego Marquez, Alison R. Howarth, Emily Dema, Fiona Burns, Andrew J. Copas, Catherine H. Mercer, Pam Sonnenberg, Kirstin R. Mitchell, Nigel Field, Jo Gibbs

**Affiliations:** 1 Institute for Global Health, 4919 University College London, London, United Kingdom; 2 Royal Free London NHS Foundation Trust, London, United Kingdom; 3 MRC/CSO Social and Public Health Sciences Unit, School of Health and Wellbeing, 3526 University of Glasgow, Glasgow, United Kingdom

**Keywords:** Screening, epidemiology, Europe

## Abstract

**Background:**

During the COVID-19 pandemic, online sexual health service delivery increased across Britain. We investigated inequalities in STI testing access and methods of access during the first year of the pandemic.

**Methods:**

Natsal-COVID, an online-survey of people 18–59 years in Britain, explored sexual health experiences in the first year of the pandemic. We describe the socio-demographics of participants who used STI testing services and compare those who reported being “online service users”, defined as using services with no direct clinician interactions (regardless of whether they also used other methods), with those who were exclusively “other service users”, defined as face-to-face, telephone, or video calls.

**Results:**

246/6,064 participants (4.2%) reported STI testing between 03/2020–03/2021. Of those, 35.8% (95%CI 29.2–42.8) used online services. Online service users (compared to other service users) were more often white (74.9% (62.2–84.4) versus 68.5% (59.4–76.3)), less often had anxiety (39.0% (28.4–50.9) versus 57.2% (48.4–65.6)) and less often had disabilities (25.8% (16.8–37.4) versus 48.1% (39.4–56.9)). Among women (only), online users were more often in good health (91.4% (81.3–96.2) versus 69.3% (57.4–79.2)).

**Conclusions:**

More than one third of STI testers used online services during this period. Differences exist in the characteristics of people accessing online versus other testing services. These data suggest that online services were more likely to be accessed by groups with typically lower risk of poor sexual health (white and in good health). Further investigation is needed, especially if online services are the only option offered, as differences in ability to access services could widen inequalities.

## Introduction

Sexual health service users without complex needs are now routinely being directed to online care and remote management and, following the pandemic, online testing continues to be the only testing option available to asymptomatic patients in many services across Britain.^1,2^ This rapid growth in online sexual health services was seen prior to the pandemic, with internet based STI screening growing by 69% (from 248,184 to 419,046) between 2018 and 2019.^3,4^ During and following the pandemic, there was a substantial increase in total recorded consultations at sexual health services, which is primarily due to a 19% increase in online consultations from 513,613 in 2019 to around 1.2 million in 2020 and 1.7 million in 2022.^2^

The use of online testing options like online postal self-sampling (OPSS) has become embedded as part of routine access to STI testing in many areas within Britain.^3,4^ OPSS allows patients to order a kit online, take their own sample, mail their samples using pre-paid postage, and receive their results online and/or via text message.^3^

Evidence as to which groups benefit most from online testing options is lacking, although there is concern that it might systematically exclude some groups of service users, including, importantly, those unable or unwilling to engage with online care.^3^ Some patterns regarding which groups access online services more frequently have already emerged. For example, OPSS users tend to be more frequently female, of white ethnicity, and live in less deprived areas,^3,5–7^ despite these groups being less affected by STIs. The question of whether the shift to remote online care is affecting equitable access to sexual healthcare at a population level has not yet been fully answered. The pandemic heralded a significant shift to remote care, and therefore provides important data to inform this question.

In 2021, the Natsal-COVID study used a web panel survey to investigate, among other issues, patterns in sexual health service use during the COVID-19 pandemic.^8^ Here, we used data from Natsal-COVID to investigate the characteristics of those who accessed STI testing and how they accessed services during the first year of the COVID-19 pandemic.

## Methods

### Study design

Natsal-COVID Wave 2 was a cross-sectional, quasi-representative web-panel survey of sexual health across Britain.^9,10^ It was administered in March-April 2021, a year after the start of first UK lockdown, using a short (on average 13-minute) online questionnaire conducted by survey research company Ipsos.^8^ Recruitment methods are detailed elsewhere.^9,10^

### Participants

Participants were members of a web panel, aged 18–59 years and resident in Britain. Quota-based sampling was used to achieve a quasi-representative sample of the general British population. Data were weighted to match general population distributions for gender, age, region, social grade, and sexual identity (Appendix 1).^10^ Participants who reported one or more sexual partners ever (hereon ‘sexually experienced participants’) were included in this analysis. Those reporting their most recent sexual partner as more than a year ago were included as they may still have required STI testing, for example, if they developed symptoms or received notification of an STI from a previous partner.

### Variables of interest

Participants reported service use using a multiple answer question. The full questionnaire is published at: https://www.natsal.ac.uk/projects/natsal-covid/. The primary outcomes explored were: (1) the reported use of any STI testing service; and (2) the reported use of online STI testing services and other types of STI testing services, defined as follows:

1. Online service users: Those who reported using any form of STI testing services where users do not interact directly with clinicians live. These were described as ‘other online services’ in the survey, e.g., OPSS. This category excluded those who used video calls. Online service users could also report using other services.
2. Other service users: Those who only reported using services where users interact directly with clinicians and were not online services (for example, face-to-face consultations, telephone, or video calls). These services could be used in any combination.

Sociodemographic, relationship, employment, and health-related variables were assessed for their association with STI testing behaviours and methods of accessing STI testing.

### Analysis

We conducted a descriptive analysis exploring use of STI testing among survey participants, using chi-square tests, according to participant characteristics. A logistic regression model was used to investigate the associations between each of these variables and STI testing uptake, and a multivariate logistic regression model was developed using variables that showed a statistically significant relationship (*p* < 0.05) with STI testing uptake in the crude logistic regression model.

In a descriptive analysis, using chi-square tests, we compared the characteristics of online service users and other service users. As a study of the general population, the sample of STI testing service users was not large enough to explore these associations through multivariable analysis.

Analyses were conducted using Stata version 17. Statistical significance was considered as *p* < 0.05. Due to differences in testing access patterns and STI risk behaviours by gender,^3,9,11^ all analyses were presented for all participants as well as stratified by gender for men (including trans men) and women (including trans women). Participants who identified in another way (*n* = 18) were included in estimates presented for all but not in gender stratified estimates.

All percentages and denominators reported in this paper have been weighted as previously described by Dema et al.^10^ Numerators reported in tables are unweighted.

## Results

Among 6,658 participants, 91.1% (95% confidence interval 90.3–91.8) reported being sexually experienced and hence are the sample for this paper. After weighting, 49.7% (48.3–51.0) were women, 87.1% (86.0–88.1) were of white ethnicity, 12.4% (11.5–13.3) were aged under 25, 96.2% (95.8–96.5) identified as heterosexual and 53.1% (51.7–54.4) were lower middle class or skilled working class. Nearly 70% (68.5–71.0) reported being in good or very good health and 45.0% (43.6–46.3) were educated to degree level (Table 1).

**Table 1.** Demographics and health characteristics of sexually experienced survey participants^a^.

Denominators (unweighted, weighted)	All participants		Men		Women	
	6072, 6064		2840, 3033		3214, 3011	
	*n*	Weighted % (95% CI)	*n*	Weighted % (95% CI)	*n*	Weighted % (95% CI)
Age						
18–24	793	12.4 (11.5–13.3)	371	13.6 (12.2–15.1)	417	11.0 (9.97–12.2)
25–29	912	13.5 (12.7–14.5)	363	12.4 (11.2–13.8)	546	14.6 (13.5–15.9)
30–34	680	10.7 (9.90–11.6)	244	9.48 (8.30–10.8)	433	11.9 (10.8–13.1)
35–44	1484	25.3 (24.1–26.5)	712	27.0 (25.2–28.9)	767	23.5 (22.0–25.1)
45–59	2203	38.1 (36.8–39.5)	1150	37.5 (35.6–39.5)	1051	38.9 (37.1–40.7)
Gender						
Men	2840	50.0 (48.7–51.4)	–	–	–	–
Women	3214	49.7 (48.3–51.0)	–	–	–	–
Identifies in another way	18	0.32 (0.20–0.51)	–	–	–	–
Ethnicity						
White	5320	87.1 (86.0–88.1)	2456	86.6 (85.0–88.1)	2848	87.5 (86.1–88.8)
Mixed/multiple ethnicities	139	1.56 (1.30–1.88)	60	1.49 (1.13–1.98)	78	1.60 (1.27–2.03)
Asian/Asian British	353	7.17 (6.44–7.97)	182	7.54 (6.48–8.77)	171	6.84 (5.87–7.94)
Black/African/Caribbean/Black British	151	3.01 (2.54–3.57)	80	2.93 (2.32–3.69)	71	3.12 (2.44–3.99)
Other ethnic group	30	1.17 (0.78–1.76)	12	1.40 (0.77–2.52)	18	0.96 (0.57–1.59)
Education						
No qualification	299	5.15 (4.56–5.81)	152	5.65 (4.75–6.70)	147	4.69 (3.96–5.55)
Below degree	2913	49.9 (48.5–51.3)	1358	51.1 (49.0–53.1)	1548	48.8 (46.9–50.6)
Degree	2860	45.0 (43.6–46.3)	1330	43.3 (41.3–45.3)	1519	46.6 (44.7–48.4)
Social grade						
Upper middle class or middle class	1790	23.1 (22.0–24.1)	1006	23.5 (22.1–25.1)	778	22.5 (21.1–24.0)
Lower middle class or skilled working class	2800	53.1 (51.7–54.4)	1176	53.4 (51.4–55.4)	1617	52.8 (51.0–54.7)
Working class or lower level of subsistence	1482	23.9 (22.7–25.0)	658	23.1 (21.4–24.8)	819	24.6 (23.1–26.2)
Region						
England	5312	86.7 (85.7–87.6)	2492	87.0 (85.5–88.3)	2807	86.5 (85.1–87.7)
Wales	275	4.78 (4.21–5.41)	124	4.63 (3.82–5.59)	150	4.92 (4.17–5.80)
Scotland	485	8.55 (7.79–9.37)	224	8.40 (7.30–9.65)	257	8.61 (7.60–9.73)
Sexuality						
Heterosexual or straight	5337	96.2 (95.8–96.5)	2472	96.2 (95.7–96.7)	2863	96.6 (96.2–97.0)
Gay or Lesbian	281	1.79 (1.56–2.04)	194	2.37 (2.03–2.76)	83	1.07 (0.83–1.37)
Bisexual	331	1.41 (1.23–1.61)	120	0.90 (0.70–1.14)	205	1.71 (1.47–1.98)
Other	67	0.66 (0.49–0.89)	25	0.50 (0.29–0.86)	36	0.60 (0.42–0.86)
Same-sex partner in the last 5 years						
No	5551	96.3 (95.8–96.7)	2530	95.4 (94.6–96.1)	3003	97.2 (96.6–97.7)
Yes	432	3.71 (3.28–4.19)	263	4.64 (3.94–5.45)	169	2.79 (2.32–3.36)
Relationship status						
Married or in a steady relationship	4301	71.5 (70.2–72.7)	1953	69.2 (67.3–71.1)	2338	73.8 (72.2–75.4)
In a new or casual relationship	279	4.86 (4.28–5.51)	135	5.44 (4.54–6.50)	142	4.24 (3.55–5.05)
Not currently in a relationship or at the end of a relationship (e.g., separating)	1401	22.7 (21.6–23.9)	699	24.2 (22.5–26.0)	699	21.2 (19.8–22.8)
In more than one type of relationship	14	0.24 (0.12–0.45)	6	0.30 (0.12–0.75)	8	0.17 (0.01–0.39)
Other	43	0.73 (0.53–1.00)	23	0.80 (0.51–1.24)	18	0.58 (0.36–0.94)
Cohabitation status						
Married/in a steady relationship and cohabitating	3761	63.0 (61.7–64.3)	1742	61.7 (59.6–63.7)	2013	64.5 (62.8–66.2)
Married/in a steady relationship and not cohabitating	540	8.47 (7.75–9.25)	211	7.57 (6.56–8.74)	325	9.28 (8.30–10.4)
Not in a steady relationship	1737	28.5 (27.3–29.8)	863	30.8 (28.9–32.7)	867	26.2 (24.6–27.9)
Employment status						
Employed	4306	71.4 (70.2–72.6)	2174	77.0 (75.3–78.7)	2125	66.0 (64.2–67.7)
Employed but on paid leave (including furlough)	330	5.18 (4.61–5.81)	130	4.48 (3.70–5.41)	198	5.85 (5.06–6.75)
Unemployed	643	10.8 (9.93–11.6)	325	11.6 (10.3–13.0)	314	9.85 (8.80–11.0)
Student	285	4.09 (3.60–4.65)	101	3.35 (2.70–4.14)	180	4.72 (4.02–5.54)
Other (incl retired, homemaker, etc.)	508	8.55 (7.83–9.33)	110	3.57 (2.92–4.37)	397	13.6 (12.4–14.9)
Became unemployed since the first lockdown						
No	5564	93.4 (92.7–94.1)	2609	93.6 (92.5–94.6)	2940	93.2 (92.3–94.1)
Yes	414	6.59 (5.94–7.31)	179	6.39 (5.44–7.49)	233	6.77 (5.91–7.74)
Furloughed under coronavirus job retention scheme						
No	5086	85.2 (84.2–86.2)	2368	84.7 (83.1–86.2)	2705	85.7 (84.4–87.0)
Yes	892	14.8 (13.9–15.8)	420	15.3 (13.8–16.9)	468	14.3 (13.1–15.6)
Currently smokes cigarettes						
No	4629	75.9 (74.7–77.1)	2066	71.7 (69.8–73.6)	2553	81.3 (78.7–81.7)
Yes	1406	24.1 (22.9–25.3)	753	28.3 (26.4–30.2)	645	19.8 (18.3–21.3)
Number of days drinking in the last week						
0 days	2318	38.2 (36.8–39.5)	910	31.9 (30.0–33.8)	1400	44.4 (42.6–46.3)
1–2 days	2217	37.2 (35.9–38.5)	1043	38.5 (36.5–40.6)	1168	35.9 (34.1–37.6)
3–4 days	940	15.3 (14.4–16.3)	531	18.0 (16.5–19.6)	405	12.6 (11.4–13.8)
5–7 days	582	9.35 (8.58–10.2)	349	11.6 (10.4–12.9)	233	7.15 (6.26–8.14)
Depression (PHQ2 score)						
No symptoms of depression (0-2)	4030	67.8 (66.5–69.1)	1905	67.3 (65.4–69.3)	2119	68.4 (66.7–70.1)
Symptoms of depression (3-6)	1943	32.2 (30.9–33.5)	888	32.7 (30.7–34.7)	1044	31.6 (29.9–33.3)
Anxiety (GAD2 score)						
No symptoms of anxiety (0-2)	4057	68.6 (67.3–69.9)	2012	71.1 (69.1–72.9)	2042	66.5 (64.8–68.2)
Symptoms of anxiety (3-6)	1953	31.4 (30.1–32.7)	792	29.0 (27.1–30.9)	1148	33.5 (31.8–35.2)
General health						
Bad/very bad	406	6.46 (5.83–7.17)	174	5.83 (4.96–6.85)	229	7.03 (6.13–8.04)
Fair	1433	23.8 (22.6–25.0)	665	24.1 (22.4–25.9)	760	23.3 (21.8–24.9)
Good/very good	4216	69.8 (68.5–71.0)	1991	70.1 (68.2–71.9)	2219	69.7 (68.0–71.4)
Disability						
None	3885	66.4 (65.1–67.7)	1917	69.8 (67.8–71.7)	1966	63.4 (61.6–65.1)
Yes, not limiting	550	8.63 (7.91–9.42)	233	7.51 (6.53–8.62)	317	9.82 (8.78–11.0)
Yes, limiting	1534	25.0 (23.8–26.2)	638	22.7 (21.0–24.5)	882	26.8 (25.2–28.5)

CI: Confidence Intervals.

^a^Some variables have instances of missing values not shown in table 1 (<2% in all cases). All percentages presented are weighted and all numerators are unweighted.

### Use of STI testing services

In total, a weighted prevalence of 4.2% (3.7–4.8) of participants reported using any STI testing service between March 2020 and March 2021 (Table 2).

**Table 2.** STI testing service use among sexually experienced participants^a^.

	All							Men							Women						
	No service use, Weighted *N* = 5648			Any type of STI testing service, Weighted *N* = 246			*p*-value	No service use, Weighted *N* = 2825			Any type of STI testing service, Weighted *N* = 121			*p*-value	No service use, Weighted *N* = 2806			Any type of STI testing service, Weighted *N* = 124			*p*-value
	*n*	%^b^	95% CI	*n*	%^b^	95% CI		*n*	%^b^	95% CI	*n*	%^b^	95% CI		*n*	%^b^	95% CI	*n*	%^b^	95% CI	
Age																					
18–24	651	10.9	(10.0–11.9)	101	38.4	(32.0–45.3)	<0.001	313	12.4	(11.0–14.0)	38	34.9	(25.6–45.6)	<0.001	334	9.32	(8.31–10.4)	63	42.2	(33.6–51.2)	<0.001
25–29	810	12.8	(11.9–13.8)	77	28.7	(22.9–35.3)		320	11.7	(10.4–13.1)	31	26.6	(18.4–36.8)		488	14.0	(12.8–15.3)	45	30.1	(22.5–39.0)	
30–34	631	10.6	(9.78–11.5)	37	14.3	(10.2–19.8)		221	9.24	(8.04–10.6)	17	15.3	(9.06–24.7)		407	11.9	(10.8–13.2)	20	13.5	(8.72–20.3)	
35–44	1403	25.7	(24.4–26.9)	39	13.0	(9.20–18.0)		668	27.2	(25.4–29.2)	24	16.5	(10.4–25.1)		730	24.0	(22.5–25.7)	15	9.70	(5.78–15.8)	
45–59	2149	40.0	(38.6–41.4)	22	5.55	(3.35–9.07)		1120	39.5	(37.4–41.5)	17	6.65	(3.61–11.9)		1027	40.7	(38.8–42.7)	5	4.53	(1.88–10.5)	
Gender																					
Men	2642	50.0	(48.6–51.4)	127	49.0	(42.2–55.8)	0.907	–	–	–	–	–	–	–	–	–	–	–	–	–	–
Women	2986	49.7	(48.3–51.1)	148	50.6	(43.8–57.3)		–	–	–	–	–	–		–	–	–	–	–	–	
Identifies in another way	16	0.31	(0.19–0.50)	1	0.44	(0.00–3.08)		–	–	–	–	–	–		–	–	–	–	–	–	
Ethnicity																					
White	4999	88.1	(87.0–89.0)	203	69.2	(62.0–75.5)	<0.001	2316	87.9	(86.3–89.3)	85	64.4	(53.3–74.1)	<0.001	2668	88.2	(86.8–89.5)	118	74.3	(64.8–82.0)	<0.001
Mixed/multiple ethnicities	116	1.39	(1.14–1.70)	19	5.34	(3.20–8.77)		48	1.29	(0.94–1.76)	10	6.33	(3.11–12.5)		68	1.51	(1.17–1.94)	8	3.53	(1.62–7.53)	
Asian/Asian British	322	6.94	(6.20–7.77)	17	9.04	(5.55–14.4)		164	7.22	(6.15–8.47)	12	12.8	(7.04–22.0)		158	6.71	(5.72–7.85)	5	5.58	(2.32–12.8)	
Black/African/Caribbean/Black British	124	2.68	(2.22–3.23)	23	11.2	(7.39–16.6)		66	2.66	(2.05–3.43)	11	9.19	(5.00–16.3)		58	2.71	(2.06–3.57)	12	13.2	(7.55–2.21)	
Other ethnic group	23	0.91	(0.58–1.44)	6	5.27	(2.23–11.9)		8	0.95	(0.47–1.94)	3	7.34	(2.37–20.5)		15	0.88	(0.50–1.53)	3	3.34	(1.00–10.6)	
Sexuality																					
Heterosexual or straight	5012	96.4	(96.0–96.8)	202	89.8	(86.3–92.4)	<0.001	2340	96.7	(96.2–97.2)	80	85.0	(78.4–89.8)	<0.001	2670	96.7	(96.2–97.1)	122	95.1	(92.4–96.8)	0.022
Gay or lesbian	234	1.63	(1.41–1.89)	40	5.68	(4.00–8.01)		155	2.05	(1.72–2.43)	35	10.7	(7.22–15.6)		75	1.07	(0.82–1.39)	5	1.04	(0.41–2.63)	
Bisexual	295	1.28	(1.12–1.46)	28	3.94	(2.27–6.74)		110	0.78	(0.63–0.97)	6	3.08	(0.96–9.49)		181	1.63	(1.40–1.91)	21	3.89	(2.40–6.25)	
Other	63	0.67	(0.50–0.91)	2	0.61	(0.15–2.43)		21	0.46	(0.25–0.84)	2	1.26	(0.31–4.99)		36	0.65	(0.45–0.93)	0	0	––	
Cohabitation status																					
Married/steady relationship and cohabitating	3585	64.2	(62.9–65.6)	98	35.9	(29.6–42.8)	<0.001	1649	62.2	(60.1–64.3)	56	45.5	(35.4–56.1)	0.005	1931	66.4	(64.6–68.2)	41	26.2	(19.1–34.8)	<0.001
Married/steady relationship and not cohabitating	472	8.01	(7.29–8.80)	45	15.7	(11.5–20.9)		189	7.35	(6.31–8.55)	16	12.3	(7.11–20.4)		279	8.56	(7.59–9.64)	29	19.1	(13.2–26.7)	
Not in a steady relationship	1568	27.8	(26.5–29.1)	129	48.4	(41.6–55.2)		791	30.4	(28.5–32.4)	52	42.2	(32.3–52.8)		770	25.0	(23.4–26.7)	77	54.7	(45.8–63.4)	
Education																					
No qualification	273	5.08	(4.47–5.76)	14	5.82	(3.25–10.2)	0.808	141	5.70	(4.77–6.81)	7	5.45	(2.41–11.9)	0.808	132	4.48	(3.75–5.35)	7	6.22	(2.72–13.6)	0.513
Below degree	2690	49.7	(48.3–51.1)	136	5.09	(44.1–57.7)		1258	50.8	(48.7–52.9)	56	49.7	(39.5–60.0)		1426	48.6	(46.7–50.5)	79	51.6	(42.7–60.4)	
Degree	2681	45.3	(43.9–46.7)	126	43.3	(36.7–50.1)		1243	43.5	(41.4–45.6)	64	44.8	(34.9–55.1)		1428	46.9	(45.0–48.8)	62	42.2	(33.7–51.2)	
Social grade																					
Upper middle class or middle class	1684	24.0	(22.3–24.5)	83	24.1	(19.2–29.9)	0.483	948	24.0	(22.5–25.5)	46	24.5	(17.5–33.3)	0.991	731	22.8	(21.3–24.4)	36	23.0	(16.7–31.0)	0.212
Lower middle class or skilled working class	2611	53.1	(51.7–54.5)	117	49.3	(42.6–56.1)		1084	53.1	(51.0–55.1)	56	52.7	(42.5–62.8)		1520	53.2	(51.3–55.1)	61	46.5	(37.7–55.5)	
Working class or lower level of subsistence	1349	23.5	(22.4–24.7)	76	26.5	(21.0–32.9)		610	23.0	(21.3–24.8)	25	22.8	(15.1–32.8)		735	24.0	(22.4–25.7)	51	30.5	(23.1–39.0)	
Region																					
England	4931	86.6	(85.6–87.5)	253	91.2	(86.5–94.4)	0.159	2313	86.9	(85.3–88.3)	118	91.5	(83.4–95.8)	0.449	2606	86.4	(85.0–87.7)	134	90.9	(84.3–94.8)	0.367
Wales	260	4.83	(4.25–5.50)	8	3.41	(1.63–7.01)		118	4.69	(3.85–5.70)	3	3.24	(1.05–9.59)		141	4.97	(4.19–5.89)	5	3.61	(1.36–9.25)	
Scotland	453	8.59	(7.80–9.44)	15	5.37	(3.00–9.46)		211	8.46	(7.33–9.75)	6	5.25	(2.03–12.9)		239	8.65	(7.61–9.82)	9	5.54	(2.71–11.0)	
Same sex partner in last 5 years																					
No	5218	96.9	(96.4–97.3)	201	82.9	(77.0–87.5)	<0.001	2400	96.3	(95.6–96.9)	73	72.6	(62.2–81.1)	<0.001	2802	97.4	(96.9–97.9)	127	92.3	(86.8–95.5)	<0.001
Yes	357	3.14	(2.75–3.58)	65	17.1	(12.5–23.0)		211	3.71	(3.11–4.43)	46	27.4	(19.0–37.8)		146	2.58	(2.12–3.15)	19	7.75	(4.46–13.2)	
Employment status																					
Employed	4004	71.4	(70.1–72.6)	196	71.8	(65.4–77.5)	<0.001	2027	77.2	(75.4–78.9)	97	75.0	(65.0–82.9)	0.105	1970	65.7	(63.8–67.5)	99	69.4	(60.8–76.8)	<0.001
Employed but on paid leave (incl. furlough)	303	5.14	(4.56–5.79)	19	6.61	(3.93–10.9)		118	4.39	(3.60–5.34)	8	5.95	(2.45–13.8)		183	5.85	(5.03–6.79)	11	7.30	(3.94–13.2)	
Unemployed	602	10.8	(9.94–11.7)	21	8.64	(5.42–13.5)		302	11.5	(10.2–12.9)	13	12.4	(6.90–21.3)		297	10.0	(8.93–11.2)	7	4.19	(1.92–8.91)	
Student	239	3.69	(3.21–4.25)	33	11.1	(7.66–15.8)		88	3.15	(2.51–3.97)	8	6.43	(3.09–12.9)		148	4.14	(3.47–4.94)	25	15.7	(10.3–23.2)	
Other (incl. retired or homemaker)	496	9.01	(8.24–9.83)	7	1.83	(0.78–4.25)		107	3.75	(3.05–4.59)	1	0.20	(0.00–1.40)		388	14.3	(13.0–15.7)	6	3.43	(1.40–8.15)	
Became unemployed since first lockdown																					
No	5206	93.8	(93.1–94.4)	232	84.0	(77.8–88.7)	<0.001	2447	94.1	(93.0–95.0)	107	83.9	(73.3–90.9)	0.001	2744	93.5	(92.5–94.4)	125	84.8	(77.2–90.3)	<0.001
Yes	367	6.20	(5.56–6.92)	40	16.0	(11.3–22.2)		157	5.91	(4.99–6.98)	17	16.1	(9.12–26.7)		209	6.50	(5.63–7.49)	22	15.2	(9.75–22.9)	
Furloughed under coronavirus job retention scheme																					
No	4753	85.3	(84.2–86.3)	217	80.2	(74.2–85.1)	0.047	2217	84.7	(83.1–86.2)	98	79.5	(69.5–86.9)	0.200	2524	85.9	(84.5–87.2)	118	80.7	(73.1–86.6)	0.098
Yes	820	14.7	(13.7–15.8)	55	19.8	(14.9–25.8)		387	15.3	(13.8–16.9)	26	20.5	(13.1–30.5)		429	14.1	(12.8–15.5)	29	19.3	(13.4–26.9)	
Currently smokes cigarettes																					
No	4360	76.8	(75.5–78.0)	173	61.2	(54.1–67.8)	<0.001	1954	72.9	(71.0–74.8)	72	50.2	(39.8–60.5)	<0.001	2397	80.8	(79.2–82.2)	101	72.2	(63.5–79.5)	0.021
Yes	1260	23.2	(22.0–24.5)	97	38.8	(32.2–45.9)		674	27.1	(25.2–29.0)	52	49.8	(39.5–60.2)		579	19.3	(17.8–20.8)	44	27.8	(20.5–36.5)	
Number of days drinking in the last week																					
0 days	2195	38.8	(37.4–40.2)	66	22.90	(17.8–29.0)	<0.001	851	32.1	(30.2–34.1)	32	24.0	(16.5–33.5)	0.191	1337	45.5	(43.6–47.4)	34	22.1	(15.7–30.2)	<0.001
1–2 days	2020	36.4	(35.1–37.8)	133	49.2	(42.4–56.0)		959	38.0	(36.0–40.1)	55	44.3	(34.3–54.8)		1056	34.9	(33.1–36.7)	77	53.5	(44.6–62.1)	
3–4 days	868	15.3	(14.3–16.3)	50	19.0	(14.0–25.2)		492	17.9	(16.4–19.6)	28	23.1	(15.2–33.6)		372	12.5	(11.3–13.8)	22	15.2	(9.94–22.5)	
5–7 days	549	9.50	(8.70–10.4)	26	8.90	(5.78–13.5)		335	11.9	(10.7–13.4)	11	8.61	(4.37–16.3)		214	7.10	(6.19–8.13)	15	9.26	(5.31–15.7)	
General health																					
Bad/very bad	383	6.55	(5.89–7.29)	10	3.49	(1.70–7.03)	0.093	167	5.95	(5.04–7.01)	3	2.89	(0.78–10.2)	0.425	213	7.08	(6.15–8.14)	7	4.11	(1.81–9.04)	0.153
Fair	1333	23.7	(22.5–25.0)	57	20.5	(15.3–26.8)		619	23.9	(22.1–25.7)	23	21.2	(13.4–32.0)		707	23.5	(21.9–25.1)	33	19.0	(13.2–26.6)	
Good/very good	3918	69.7	(68.4–71.0)	206	76.0	(69.5–81.5)		1850	70.2	(68.2–72.1)	99	75.9	(65.0–84.2)		2062	69.5	(67.7–71.2)	107	76.9	(68.9–83.3)	
Depression (PHQ2 score)																					
No symptoms of depression (0–2)	3818	69.0	(67.6–70.3)	142	50.6	(43.6–57.6)	<0.001	1811	68.7	(66.7–70.7)	63	47.1	(36.8–57.6)	<0.001	2001	69.4	(67.6–71.1)	79	54.5	(45.4–63.4)	0.001
Symptoms of depression (3–6)	1756	31.0	(29.7–32.4)	121	49.4	(42.4–56.4)		797	31.3	(29.3–33.3)	58	53.0	(42.4–63.3)		949	30.6	(28.9–32.4)	62	45.5	(36.6–54.6)	
Anxiety (GAD2 score)																					
No symptoms of anxiety (0–2)	3847	70.1	(68.7–71.3)	130	48.6	(41.8–55.4)	<0.001	1912	72.9	(70.9–74.7)	64	49.4	(39.2–59.7)	<0.001	1932	67.5	(65.8–69.3)	66	48.2	(39.3–57.2)	<0.001
Symptoms of anxiety (3–6)	1747	30.0	(28.7–31.3)	142	51.4	(44.6–58.2)		698	27.2	(25.3–29.1)	62	50.6	(40.3–60.8)		1037	32.5	(30.7–34.3)	79	51.8	(42.8–60.7)	
Disability																					
None	3645	66.9	(65.5–68.2)	152	57.3	(50.3–63.9)	0.003	1812	70.8	(68.8–72.6)	68	52.9	(42.4–63.2)	0.001	1831	63.2	(61.4–65.1)	84	61.8	(52.9–70.0)	0.343
Yes, not limiting	514	8.69	(7.94–9.50)	22	7.47	(4.53–12.1)		213	7.40	(6.40–8.54)	12	8.27	(4.04–16.2)		301	10.0	(8.95–11.2)	10	6.78	(3.36–13.2)	
Yes, limiting	1405	24.5	(23.3–25.7)	96	35.3	(29.0–42.2)		580	21.8	(20.1–23.7)	43	38.8	(29.0–49.6)		812	26.7	(25.1–28.5)	52	31.4	(23.8–40.1)	

CI: Confidence Intervals.

^a^All denominators and percentages reported are weighted. Numerators are unweighted.

^b^Column percentages in all cases.

In the adjusted model (Table 3), older participants had lower odds of accessing STI testing. Compared to white participants, those from mixed/multiple ethnic backgrounds or Black/African/Caribbean/Black British backgrounds showed higher odds of accessing STI testing (aOR (95% confidence interval) = 2.73 (1.35–5.53) and aOR = 3.01 (1.60–5.64) respectively). Gay men had increased odds of having accessed STI testing compared to heterosexual/straight men (aOR = 2.58 (1.03–6.44)). Those who had a same sex partner in the previous 5 years had increased odds of having accessed STI testing (aOR among men = 4.17 (1.84–9.46), among women = 2.82 (1.04–7.66)). These participants may have had different-sex partners within the time frame, in addition to same sex partners.

**Table 3.** Associations between sociodemographic/health characteristics and STI testing service access.

	Used STI testing services (all participants)				Used STI testing services (men)				Used STI testing services (women)			
	cOR^a^	95% CI	aOR^b^	95% CI	cOR^a^	95% CI	aOR^b^	95% CI	cOR^a^	95% CI	aOR^b^	95% CI
Age	*p* < 0.001		***p* < 0.001**		*p* < 0.001		***p* < 0.001**		*p* < 0.001		***p* < 0.001**	
18–24	Ref	Ref	Ref	Ref	Ref	Ref	Ref	Ref	Ref	Ref	Ref	Ref
25–29	0.64	(0.44–0.91)	0.92	(0.59–1.45)	0.81	(0.46–1.44)	1.03	(0.48–2.20)	0.48	(0.30–0.75)	0.67	(0.39–1.17)
30–34	0.38	(0.25–0.60)	0.59	(0.33–1.04)	0.59	(0.30–1.18)	0.87	(0.35–2.17)	0.25	(0.14–0.43)	**0.41**	**(0.20–0.82)**
35–44	0.14	(0.09–22.2)	**0.29**	**(0.17–0.51)**	0.22	(0.12–0.40)	0.43	(0.18–1.03)	0.09	(0.05–16.5)	**0.16**	**(0.08–0.33)**
45–59	0.04	(0.02–0.07)	**0.09**	**(0.04–0.17)**	0.06	(0.03–0.12)	**0.13**	**(0.05–0.33)**	0.02	(0.01–0.06)	**0.05**	**(0.02–0.14)**
Gender		*p* = 0.907										
Men	Ref	Ref	–	–	–	–	–	–	–	–	–	–
Women	1.04	(0.79–1.37)	–	–	–	–	–	–	–	–	–	–
Identifies in another way	1.47	(0.19–11.2)	–	–	–	–	–	–	–	–	–	–
Ethnicity	*p* < 0.001			***p* = 0.001**	*p* < 0.001		*p* = 0.050		*p* < 0.001		*p* = 0.069	
White	Ref	Ref	Ref	Ref	Ref	Ref	Ref	Ref	Ref	Ref	Ref	Ref
Mixed/multiple ethnicities	4.87	(2.74–8.65)	**2.73**	**(1.35–5.53)**	6.71	(2.96–15.20)	**4.12**	**(1.36–12.5)**	2.78	(1.20–6.44)	1.52	(0.57–4.02)
Asian/Asian British	1.66	(0.96–2.86)	1.18	(0.59–2.34)	2.41	(1.21–4.80)	1.84	(0.77–4.42)	0.99	(0.39–2.50)	0.61	(0.20–1.88)
Black/African/Caribbean/Black British	5.33	(3.23–8.78)	**3.01**	**(1.60–5.64)**	4.72	(2.31–9.65)	**2.86**	**(1.07–7.62)**	5.78	(2.91–11.5)	**3.08**	**(1.30–7.29)**
Other ethnic group	7.35	(2.69–20.1)	2.30	(0.83–6.34)	10.49	(2.61–42.12)	0.13	(0.05–0.33)	4.52	(1.16–17.6)	1.87	(0.41–8.44)
Sexuality	*p* < 0.001		*p* = 0.223		*p* < 0.001		***p* = 0.009**		*p* = 0.004		*p* = 0.169	
Heterosexual or straight	Ref	Ref	Ref	Ref	Ref	Ref	Ref	Ref	Ref	Ref	Ref	Ref
Gay or lesbian	3.73	(2.50–5.56)	1.58	(0.78–3.17)	5.95	(3.72–9.51)	**2.58**	**(1.03–6.44)**	0.99	(0.37–2.63)	0.26	(0.06–1.12)
Bisexual	3.30	(1.84–5.92)	0.72	(0.36–1.42)	4.48	(1.33–15.15)	0.22	(0.04–1.10)	2.42	(1.43–4.09)	0.66	(0.31–1.41)
Other	0.97	(0.23–4.09)	0.68	(0.15–3.06)	3.10	(0.66–14.45)	1.75	(0.42–7.25)	–	–	–	–
Cohabitation status	*p* < 0.001		***p* < 0.001**		*p* = 0.007		*p* = 0.470		*p* < 0.001		***p* < 0.001**	
Married/steady relationship and cohabitating	Ref	Ref	Ref	Ref	Ref	Ref	Ref	Ref	Ref	Ref	Ref	Ref
Married/steady relationship and not cohabitating	3.50	(2.32–5.27)	**2.42**	**(1.47–3.98)**	2.28	(1.18–4.42)	1.64	(0.71–3.82)	5.64	(3.30–9.65)	**3.87**	**(2.08–7.19)**
Not in a steady relationship	3.11	(2.28–4.26)	**2.47**	**(1.72–3.54)**	1.89	(1.19–3.01)	1.27	(0.72–2.25)	5.55	(3.58–8.58)	**4.88**	**(3.02–7.89)**
Education	*p* = 0.808				*p* = 0.966				*p* = 0.515			
No qualification	Ref	Ref	–	–	Ref	Ref	–	–	Ref	Ref	–	–
Below degree	0.89	(0.47-1.69)	–	–	1.02	(0.42-2.51)	–	–	0.77	(0.31-1.88)	–	–
Degree	0.84	(0.44-1.59)	–	–	1.08	(0.44-2.63)	–	–	0.65	(0.26-1.61)	–	–
Social grade	*p* = 0.520				*p* = 0.990				*p* = 0.230			
Upper middle class or middle class	Ref	Ref	–	–	Ref	Ref	–	–	Ref	Ref	–	–
Lower middle class or skilled working class	0.90	(0.65-1.25)	–	–	0.97	(0.61-1.55)	–	–	0.86	(0.55-1.36)	–	–
Working class or lower level of subsistence	1.10	(0.76-1.58)	–	–	0.97	(0.54-1.72)	–	–	1.25	(0.78-2.02)	–	–
Region	*p* = 0.164				*p* = 0.465				*p* = 0.356			
England	Ref	Ref	–	–	Ref	Ref	–	–	Ref	Ref	–	–
Wales	0.67	(0.31-1.45)	–	–	0.66	(0.20-2.12)	–	–	0.69	(0.25-1.91)	–	–
Scotland	0.59	(0.32-1.10)	–	–	0.59	(0.22-1.59)	–	–	0.61	(0.29-1.30)	–	–
Same sex partner in last 5 years	*p* < 0.001		***p* < 0.001**		*p* < 0.001		***p* = 0.001**		*p* < 0.001		***p* = 0.042**	
No	Ref	Ref	Ref	Ref	Ref	Ref	Ref	Ref	Ref	Ref	Ref	Ref
Yes	6.37	(4.30–9.46)	**3.19**	**(1.74–5.83)**	9.77	(5.86–16.31)	**4.17**	**(1.84–9.46)**	3.17	(1.70–5.91)	**2.82**	**(1.04–7.66)**
Employment status	*p* < 0.001		*p* = 0.120		*p* = 0.015		*p* = 0.257		*p* < 0.001		*p* = 0.168	
Employed	Ref	Ref	Ref	Ref	Ref	Ref	Ref	Ref	Ref	Ref	Ref	Ref
Employed but on paid leave (incl furlough)	1.28	(0.72–2.26)	1.02	(0.51–2.02)	1.40	(0.54–3.63)	0.59	(0.14–2.48)	1.18	(0.60–2.34)	1.45	(0.69–3.04)
Unemployed	0.80	(0.48–1.33)	**0.48**	**(0.25–0.91)**	1.11	(0.57–2.16)	0.55	(0.22–1.34)	0.40	(0.17–0.90)	0.35	(0.12–1.00)
Student	2.98	(1.92–4.63)	0.90	(0.50–1.60)	2.10	(0.93–4.72)	0.70	(0.23–2.18)	3.59	(2.12–6.09)	0.91	(0.46–1.78)
Other (incl retired, homemaker...)	0.20	(0.08–0.49)	0.48	(0.20–1.18)	0.05	(0.01–0.39)	0.17	(0.02–1.27)	0.23	(0.09–0.58)	0.59	(0.23–1.53)
Became unemployed since first lockdown	*p* < .001		***p* = 0.017**		*p* = 0.001		*p* = 0.182		*p* < 0.001		*p* = 0.200	
No	Ref	Ref	Ref	Ref	Ref	Ref	Ref	Ref	Ref	Ref	Ref	Ref
Yes	2.88	(1.89–4.38)	**1.79**	**(1.11–2.88)**	3.05	(1.56–5.95)	1.71	(0.78–3.76)	2.57	(1.52–4.36)	1.52	(0.80–2.87)
Currently smokes cigarettes	*p* < 0.001		*p* = 0.413		*p* < 0.001		*p* = 0.120		*p* = 0.022		*p* = 0.982	
No	Ref	Ref	Ref	Ref	Ref	Ref	Ref	Ref	Ref	Ref	Ref	Ref
Yes	2.10	(1.56–2.83)	1.16	(0.81–1.65)	2.68	(1.74–4.12)	1.54	(0.89–2.64)	1.62	(1.07–2.44)	0.99	(0.61–1.63)
Number of days drinking in the last week	*p* < 0.001		***p* = 0.006**		*p* = 0.188		*p* = 0.217		*p* < 0.001		***p* = 0.001**	
0 days	Ref	Ref	Ref	Ref	Ref	Ref	Ref	Ref	Ref	Ref	Ref	Ref
1–2 days	2.29	(1.62–3.22)	**2.00**	**(1.34–2.98)**	1.56	(0.93–2.62)	1.53	(0.81–2.91)	3.15	(2.00–4.97)	**2.52**	**(1.53–4.13)**
3–4 days	2.11	(1.36–3.26)	**1.98**	**(1.21–3.24)**	1.73	(0.92–3.24)	1.81	(0.84–3.88)	2.49	(1.39–4.49)	**2.01**	**(1.07–3.78)**
5–7 days	1.59	(0.93–2.70)	1.63	(0.86–3.09)	0.97	(0.43–2.18)	0.75	(0.26–2.15)	2.68	(1.34–5.37)	**3.85**	**(1.67–8.88)**
General health	*p* = 0.094				*p* = 0.429				*p* = 0.163			
Bad/very bad	Ref	Ref	–	–	Ref	Ref	–	–	Ref	Ref	–	–
Fair	1.62	(0.73-3.60)	–	–	1.84	(0.44-7.64)	–	–	1.40	(0.56-3.51)	–	–
Good/very good	2.05	(0.97-4.32)	–	–	2.23	(0.58-8.59)	–	–	1.91	(0.81-4.51)	–	–
Depression (PHQ2 score)	*p* < 0.001		*p* = 0.982		*p* < 0.001		*p* = 0.380		*p* = 0.001		*p* = 0.436	
No symptoms of depression (0-2)	Ref	Ref	Ref	Ref	Ref	Ref	Ref	Ref	Ref	Ref	Ref	Ref
Symptoms of depression (3-6)	2.17	(1.63–2.89)	1.00	(0.64–1.55)	2.47	(1.60–3.82)	1.37	(0.68–2.75)	1.89	(1.30–2.76)	0.81	(0.47–1.39)
Anxiety (GAD2 score)	*p* < 0.001		*p* = 0.611		*p* < 0.001		*p* = 0.755		*p* < 0.001		*p* = 0.311	
No symptoms of anxiety (0–2)	Ref	Ref	Ref	Ref	Ref	Ref	Ref	Ref	Ref	Ref	Ref	Ref
Symptoms of anxiety (3–6)	2.47	(1.87–3.28)	1.12	(0.73–1.72)	2.74	(1.79–4.21)	0.89	(0.44–1.80)	2.24	(1.54–3.24)	1.30	(0.78–2.17)
Disability	*p* = 0.005		***p* = 0.005**		*p* = 0.001		***p* = 0.004**		*p* = 0.347		*p* = 0.582	
None	Ref	Ref	Ref	Ref	Ref	Ref	Ref	Ref	Ref	Ref	Ref	Ref
Yes, not limiting	1.00	(0.58–1.74)	1.15	(0.61–2.16)	1.49	(0.67–3.31)	1.65	(0.61–4.46)	0.69	(0.32–1.48)	0.84	(0.38–1.88)
Yes, limiting	1.68	(1.24–2.28)	**1.87**	**(1.28–2.74)**	2.37	(1.49–3.77)	**2.67**	**(1.49–4.79)**	1.20	(0.81–1.79)	1.26	(0.75–2.14)

^a^Crude Odds Ratio.

b Multivariate-Adjusted Odds Ratio (adjusted for variables found significant in crude model).

Values in bold denote statistically significant differences between groups.

Those who were unemployed were less likely to have accessed STI testing (aOR = 0.48 (0.25–0.91)). However, those who had become unemployed since the start of the first lockdown had increased odds of having accessed STI testing (aOR = 1.79 (1.11–2.88)).

Among women, those who reported drinking at least once a week were more likely to have accessed STI testing compared to non-drinkers, with those who drank 5–7 days a week showing the strongest association with STI testing (aOR = 3.85 (1.67–8.88)). Among men, the association between drinking alcohol and STI testing was not statistically significant.

Finally, participants who reported having a limiting disability had higher odds of having accessed STI testing (aOR = 1.87 (1.28–2.74)) compared to those who did not report having a disability. In the gender disaggregated models, disability status was only significantly associated with STI testing among men (aOR = 2.67 (1.49–4.79)) and not among women.

### Methods of accessing STI testing

Among those who reported accessing STI testing in the past year (weighted *N* = 216, of which *N* = 103 men and *N* = 112 women), a weighted prevalence of 35.8% did so online and 64.3% reported using only services other than online. Among men, 30.6% were online service users, while among women 40.8% were online service users.

Of online service users, 24.7% had also used another service. The most common other service type was face-to-face (74.8%), followed by phone calls (54.0%) and video calls (19.4%). Compared to other service users, online service users more frequently identified as white (74.9% (62.2–84.4) vs. 68.5% (59.4–76.3)) (Table 4). Among women, online service users were more frequently aged 25–29 (45.6% (31.3–60.7) vs. 22.5% (14.1–33.9)) but less frequently aged below 25 (30.0% (18.4–44.9) vs. 46.7% (35.2–58.7)). There was no statistically significant association between age and mode of STI testing among men. Among men, online service users were more likely to be gay than other service users (16.3% (8.52–28.9) vs. 10.5% (6.28–17.1)). Among women, those who used online services were less frequently bisexual than those who used other services (1.03% (0.31–3.32) vs. 5.81% (3.29–10.0)).

**Table 4.** Method of STI testing service use (online or other) among participants who had accessed STI testing^b^.

	All participants (*N* = 216)					Men (*N* = 103)					Women (*N* = 112)				
	Online service^a^		Other service only		*p*-val	Online service^a^		Other service only		*p*-val	Online service^a^		Other service only		*p*-val
	%, *N* = 77	95% CI	%, *N* = 139	95% CI		%, *N* = 32	95% CI	%, *N* = 71	95% CI		%, *N* = 46	95% CI	%, *N* = 66	95% CI	
Age															
18–24	27.0	(17.6–38.9)	38.0	(30.0–46.7)	0.234	22.6	(9.50–44.8)	30.4	(20.1–43.2)	0.719	30.0	(18.4–44.9)	46.7	(35.2–58.7)	**0.008**
25–29	38.2	(27.3–50.4)	25.3	(18.4–33.7)		27.4	(13.5–47.7)	26.8	(16.8–39.8)		45.6	(31.3–60.7)	22.5	(14.1–33.9)	
30–34	15.6	(8.30–27.5)	14.8	(9.60–22.2)		25.8	(11.3–48.8)	14.5	(7.34–26.5)		8.64	(3.53–19.6)	15.5	(8.77–25.8)	
35–44	15.7	(9.44–25.0)	14.0	(8.78–21.7)		15.7	(7.17–30.9)	20.9	(12.0–33.9)		15.8	(7.87–29.1)	6.83	(2.93–15.1)	
45–59	3.49	(1.05–10.9)	7.89	(4.51–13.5)		8.55	(2.53–25.2)	7.45	(3.69–14.5)		0	––	8.50	(3.53–19.1)	
Gender															
Men	40.8	(29.5–53.2)	51.4	(42.7–60.0)	0.262	–	–	–	–	–	–	–	–	–	–
Women	59.2	(46.8–70.5)	47.8	(39.3–56.5)		–	–	–	–		–	–	–	–	
Identifies in another way	0	0	0.79	(0.11–5.43)		–	–	–	–		–	–	–	–	
Ethnicity															
White	74.9	(62.2–84.4)	68.5	(59.4–76.3)	**0.028**	69.7	(49.6–84.3)	65.8	(52.1–77.3)	0.059	78.5	(60.2–89.7)	72.4	(59.8–82.3)	0.254
Mixed/multiple ethnicities	4.57	(1.69–11.8)	6.68	(3.66–11.9)		10.8	(3.77–27.2)	5.64	(2.08–14.4)		0.29	(0.00–2.09)	6.23	(2.77–13.4)	
Asian/Asian British	0.52	(0.00–3.69)	12.9	(7.49–21.2)		1.28	(0.17–9.00)	19.8	(10.7–33.7)		0	––	5.90	(1.87–17.1)	
Black/African/Caribbean/Black British	15.2	(7.87–27.4)	10.3	(5.89–17.3)		11.8	(4.28–28.7)	8.80	(3.78–19.1)		17.5	(7.50–35.8)	11.9	(5.63–23.5)	
Other ethnic group	4.80	(1.20–17.3)	1.71	(0.36–7.73)		6.36	(0.88–34.1)	0	––		3.72	(0.52–22.4)	3.51	(0.74–15.2)	
Sexual identity															
Heterosexual or straight	88.4	(80.1–93.5)	89.9	(85.6–93.0)	0.546	74.3	(56.2–86.7)	88.2	(81.3–92.7)	0.086	98.1	(95.1–99.2)	93.1	(88.5–96.0)	**0.030**
Gay or lesbian	7.18	(4.12–12.2)	5.85	(3.66–9.20)		16.3	(8.52–28.9)	10.5	(6.28–17.1)		0.92	(0.22–3.76)	1.06	(0.25–4.35)	
Bisexual	3.39	(0.74–14.2)	4.29	(2.47–7.35)		6.82	(1.07–33.0)	1.34	(0.40–4.40)		1.03	(0.31–3.32)	5.81	(3.29–10.0)	
Other	1.07	(0.15–7.31)	0	––		2.62	(0.35–17.0)	0	––		0	––	0	––	
Relationship status															
Married or in a steady relationship	35.1	(24.9–46.9)	58.9	(50.0–67.2)	**0.001**	40.0	(22.8–60.0)	66.7	(53.6–77.7)	**0.003**	31.8	(20.1–46.3)	49.8	(38.0–61.6)	0.132
In a new or casual relationship	9.50	(4.61–18.6)	16.6	(10.7–24.8)		0	––	15.5	(7.80–28.4)		16.1	(7.85–30.0)	18.0	(10.1–30.1)	
Not currently in a relationship or at the end of a relationship (e.g., separating)	51.2	(39.3–62.9)	23.1	(16.7–30.9)		49.7	(30.6–68.9)	17.8	(10.3–28.9)		52.2	(37.5–66.5)	29.1	(19.4–41.1)	
In more than one type of relationship	2.59	(0.36–16.3)	0.62	(0.00–4.33)		6.36	(0.88–34.1)	0	––		0	––	1.30	(0.18–8.83)	
Other	1.63	(0.23–10.8)	0.88	(0.12–6.04)		4.01	(0.54–24.2)	0	––		0	––	1.84	(0.25–12.1)	
Cohabitation status															
Married/steady relationship and cohabitating	25.4	(16.6–36.8)	39.8	(31.6–48.7)	**0.005**	30.3	(15.6–50.7)	52.4	(39.6–64.9)	0.072	22.0	(12.3–36.0)	25.4	(16.6–36.7)	0.071
Married/steady relationship and not cohabitating	9.75	(4.93–18.4)	19.0	(13.3–26.6)		9.67	(2.85–28.1)	14.3	(7.43–25.7)		9.81	(4.34–20.7)	24.4	(15.8–35.8)	
Not in a steady relationship	64.9	(53.1–75.1)	41.1	(32.8–50.0)		60.0	(40.0–77.2)	33.3	(22.3–46.4)		68.2	(53.7–79.9)	50.2	(38.4–62.0)	
General health															
Bad/very bad	2.59	(0.60–10.5)	2.59	(0.93–7.01)	0.183	4.12	(0.56–24.7)	0.34	(0.00–2.46)	0.328	1.52	(0.21–10.3)	5.06	(1.70–14.1)	**0.007**
Fair	11.4	(5.40–22.5)	22.4	(15.9–30.6)		17.5	(6.05–41.2)	18.2	(9.96–30.9)		7.13	(2.85–16.7)	25.6	(16.6–37.3)	
Good/very good	86.0	(74.7–92.7)	75.0	(66.7–81.8)		78.4	(55.4–91.4)	81.5	(68.9–89.8)		91.4	(81.3–96.2)	69.3	(57.4–79.2)	
Education															
No qualification	4.75	(1.55–13.6)	3.50	(1.44–8.27)	0.908	4.80	(0.78–24.4)	5.31	(1.86–14.3)	0.558	4.72	(1.12–17.9)	1.60	(0.31–7.93)	0.306
Below degree	50.5	(38.6–62.3)	51.9	(43.2–60.4)		58.4	(38.8–76.7)	45.8	(33.4–58.8)		45.0	(31.0–59.9)	57.6	(45.5–68.8)	
Degree	44.8	(33.4–56.8)	44.7	(36.2–53.4)		36.8	(20.8–56.4)	48.9	(36.4–61.6)		50.3	(35.6–64.9)	40.8	(29.7–53.0)	
Social grade															
Upper middle class or middle class	19.9	(12.9–29.4)	27.5	(20.8–35.3)	0.273	17.2	(8.26–32.5)	27.5	(18.7–38.5)	0.485	21.7	(12.6–34.9)	26.2	(17.1–38.0)	0.394
Lower middle class or skilled working class	56.5	(44.8–67.6)	45.5	(36.9–54.4)		63.3	(44.3–78.9)	52.2	(39.6–64.6)		51.9	(37.2–66.3)	39.1	(28.0–51.4)	
Working class or lower level of subsistence	23.6	(15.2–34.7)	27.0	(20.2–35.2)		19.5	(8.71–38.0)	20.3	(12.0–32.3)		26.4	(15.4–41.4)	34.7	(24.5–46.6)	
Region															
England	93.2	(82.8–97.5)	89.7	(83.0–93.9)	0.728	93.6	(65.8–99.1)	88.5	(77.0–94.7)	0.549	92.8	(80.4–97.6)	90.8	(81.7–95.6)	0.383
Wales	2.50	(0.71–8.46)	3.45	(1.28–8.97)		0	––	5.47	(1.75–15.8)		4.23	(1.19–14.0)	1.34	(0.18–9.10)	
Scotland	4.35	(1.06–16.1)	6.87	(3.60–12.7)		6.37	(0.88–34.2)	6.04	(2.07–16.4)		2.95	(0.41–18.5)	7.87	(3.54–16.6)	
Same sex partner in last 5 years															
No	85.5	(76.8–91.3)	84.3	(77.6–89.2)	0.795	82.3	(68.4–90.9)	74.0	(62.3–83.0)	0.297	87.5	(74.5–94.3)	94.9	(88.7–97.7)	0.106
Yes	14.5	(8.73–23.2)	15.7	(10.8–22.4)		17.7	(9.08–31.7)	26.1	(17.0–37.8)		12.5	(5.66–25.5)	5.14	(2.26–11.3)	
Became unemployed since first lockdown															
No	83.6	(72.1–90.9)	85.1	(77.6–90.4)	0.798	73.3	(51.8–87.5)	90.4	(79.0–96.0)	0.054	90.7	(77.9–96.4)	80.8	(69.2–88.7)	0.157
Yes	16.4	(9.09–27.9)	14.9	(9.58–22.4)		26.8	(12.5–48.2)	9.56	(4.04–21.0)		9.32	(3.59–22.1)	19.2	(11.3–30.8)	
Furloughed under coronavirus job retention scheme															
No	77.3	(66.0–85.7)	82.7	(75.4–88.2)	0.351	73.4	(52.8–87.2)	85.7	(74.3–92.5)	0.183	80.0	(66.1–89.2)	79.3	(68.3–87.2)	0.922
Yes	22.7	(14.3–34.0)	17.3	(11.8–24.6)		26.6	(12.8.47.2)	14.3	(7.48–25.7)		20.0	(10.9–33.9)	20.7	(12.8–31.7)	
Currently smokes cigarettes															
No	72.4	(60.0-82.1)	57.1	(48.3-65.6)	**0.045**	64.5	(42.4-81.7)	46.3	(34.0-59.1)	0.155	77.6	(63.2-87.4)	69.7	(57.9-79.4)	0.354
Yes	27.6	(17.9-40.1)	42.9	(34.4-51.8)		35.5	(18.3-57.6)	53.7	(40.9-66.1)		22.4	(12.6-36.8)	30.3	(20.6-42.2)	
Number of days drinking in the last week															
0 days	15.7	(9.18–25.6)	25.3	(18.4–33.6)	0.344	17.1	(7.46–34.5)	25.8	(16.1–38.6)	0.822	14.8	(7.07–28.4)	25.1	(16.3–36.7)	0.341
1–2 days	57.2	(45.1–68.4)	45.6	(37.1–54.4)		47.9	(29.1–67.3)	43.3	(31.1–56.3)		63.6	(48.8–76.3)	47.1	(35.6–59.1)	
3–4 days	16.3	(9.11–27.6)	19.7	(13.6–27.5)		22.3	(9.42–44.1)	22.2	(13.4–34.6)		12.3	(5.54–25.0)	17.3	(9.97–28.2)	
5–7 days	10.7	(4.89–21.8)	9.49	(5.64–15.6)		12.7	(3.26–38.8)	8.74	(4.27–17.1)		9.30	(3.93–20.4)	10.5	(4.85–21.1)	
Depression (PHQ2 score)															
No symptoms of depression (0–2)	61.4	(49.0–72.5)	49.1	(40.2–58.1)	0.114	55.4	(35.4–73.8)	50.8	(37.5–63.9)	0.706	65.6	(50.0–78.4)	48.3	(36.4–60.4)	0.082
Symptoms of depression (3-6)	38.6	(27.5–51.0)	50.9	(41.9–59.8)		44.6	(26.2–65.6)	49.3	(36.1–62.5)		34.4	(21.6–50.0)	51.7	(39.7–63.6)	
Anxiety (GAD2 score)															
No symptoms of anxiety (0–2)	61.0	(49.1–71.6)	42.8	(34.4–51.6)	**0.015**	61.8	(42.0–78.4)	44.6	(32.3–57.5)	0.148	60.4	(45.5–73.5)	41.6	(30.4–53.6)	0.052
Symptoms of anxiety (3–6)	39.0	(28.4–50.9)	57.2	(48.4–65.6)		38.2	(21.6–58.0)	55.4	(42.5–67.7)		39.7	(26.5–54.5)	58.4	(46.4–69.6)	
Disability															
No	74.2	(62.6–83.2)	51.9	(43.1–60.6)	**0.003**	70.7	(49.7–85.6)	49.0	(36.3–61.9)	0.078	76.6	(62.6–86.4)	55.7	(43.7–67.0)	**0.021**
Yes	25.8	(16.8–37.4)	48.1	(39.4–56.9)		29.3	(14.4–50.4)	51.0	(38.1–63.7)		23.4	(13.6–37.4)	44.3	(33.0–56.3)	
Limiting disability															
No	29.8	(12.5––55.7)	16.0	(8.62–27.9)	0.217	27.2	(5.37–71.1)	20.4	(9.14–39.5)	0.718	32.0	(11.2–63.7)	11.5	(4.05–28.6)	0.128
Yes	70.2	(44.3–87.5)	84.0	(72.2–91.4)		72.8	(28.9–94.6)	79.6	(60.5–90.9)		68.0	(36.3–88.8)	88.5	(71.4–96.0)	

^a^Online services are defined as those where service users do not interact with a clinician live, such as online postal self-sampling. 24.7% of participants who used online services also used other services in combination.

^b^Column percentages in all cases. All percentages and denominators are weighted.

Values in bold denote statistically significant differences between groups.

Among women, online service users more frequently reported being in good or very good health when compared to other service users (91.4% (81.3–96.2) vs. 69.3% (57.4–79.2)), but this was not the case among men. Online service users less frequently reported symptoms of anxiety (39.0% (28.4–50.9) vs. 57.2% (48.4–65.6)) and, among women, depression (34.4% (21.6–50.0) vs. 51.7% (39.7–63.6) than other service users. Similarly, online service users also less often reported having a disability than other service users (25.8% (16.8–37.4) vs. 48.1% (39.4–56.9)).

## Discussion

Analyses of data collected from a survey of the British general population suggest that online service users were not typically in groups that experience the highest burden of poor sexual health. Those who were younger, from mixed ethnic backgrounds or from Black/African/Caribbean/Black British ethnic backgrounds showed higher odds of accessing STI testing generally. However, online service users were more often white, more often in good health, and less often reported symptoms of anxiety and depression when compared to other service users. These differences in users could be indicative of a wider gap in access if online services are ever the only option available to a group seeking STI testing, for example asymptomatic testers.

### General patterns in STI testing uptake during the pandemic

In line with previous studies, participants who reported having used some form of STI testing were on average younger than non-testers. Similarly, STI testers who identified as men less often reported their sexual identity as heterosexual/straight than men who had not used STI testing services.^2^ In this survey, STI testers were less frequently white than non-testers. In Britain people from some Black ethnic backgrounds such as Black Caribbean heritage have a higher incidence of STI infections. This finding may indicate that their increased STI testing needs and demand are, at least to some degree, being met even in a pandemic setting.

STI testers more often reported experiencing symptoms of depression and anxiety. This relationship could in part be due to the negative effects that acquiring or being exposed to an STI may have on mental health.^12^ Furthermore, some studies have shown an association between adverse mental health conditions and STI risk behaviours like condomless sex.^13^ This is of particular relevance as the COVID-19 pandemic led to an increase in mental health disorders and reduced access to mental health support in many settings, and particularly among young people who are also most affected by STIs.^14–17^

Finally, STI testers more often reported having a disability than non-testers. The nature of these disabilities, for example whether they were physical, mental or cognitive, was not recorded in this survey. This, combined with the small sample size of participants who had accessed STI testing, largely limits the inferences that can be made. However, previous research has found that STIs are over-represented among people with learning disabilities.^18^

### Patterns in method of accessing STI testing

Among STI testers, those who had accessed online services were less frequently aged below 25, but more frequently aged 25–29 when compared to other service users. This pattern is consistent with findings of other studies.^5^ Online services like OPSS often require that a user receive a self-sampling kit in the mail. During COVID-19 lockdowns, many young people lived with their families and may not have felt they had a private way to receive testing kits, potentially leading to lower uptake.^19,20^

As seen in previous studies,^5^ online service users more often identified as white than other service users. Further investigation is needed to determine if some ethnic groups are facing barriers in accessing online testing, particularly given that certain groups (including Black Caribbean and Black African) face a disproportionate burden of STIs.^2^

Online service users who were women (but not men) more frequently reported being in good health when compared to other service users. Similarly, online service users less frequently reported anxiety than other service users. This may be linked to experiencing symptoms of an STI,^12^ which would most likely result in referral to in-clinic testing rather than being offered online testing. Further research should explore whether those experiencing anxiety may face barriers in accessing online services, particularly given the adverse effects on mental health suffered by many during the COVID-19 pandemic.^14–17^ However, these health questions did not refer specifically to sexual health.

These results reflect that users in groups that experience the highest burden of adverse sexual health less frequently accessed online services than other groups. This includes, for example, those from minority ethnic groups like Black Caribbean communities and those with poorer physical and mental health. This could mean that these users prefer more traditional methods of care or may have more complex needs that require in-person examination. However, it could also indicate barriers in accessing online services. Furthermore, with asymptomatic care increasingly shifting online, if online services become the only way for users to seek STI testing this could become a source of widening health inequalities.

### Strengths and limitations of the analysis

Participants included in this analysis after weighting were largely representative of the wider population in Britain with regard to ethnicity, age, gender, and rurality.^11^ Participants were less likely to be married or to report being in good health than the general population.^21^ The sample included in this analysis was also better educated but otherwise had a similar social grade structure to the general population.^13–15^

The target population for Natsal-COVID was the general population rather than, for example, a clinic population. Due to this it is more comprehensive, capturing both those who tried to access sexual health services as well as those who actually did so. Additionally, since recruitment for this study was not limited to a specific sexual health service, a wide view of sexual healthcare-seeking behaviours and experiences across all types of services and service users can be presented. However, as the target population is the general population, this study includes a relatively small number of participants reporting use of STI testing services and specifically online services. Thus, caution must be taken when interpreting the results. The small sample size limited our analysis of service type to descriptive level and meant that we lacked power to provide strong statistical evidence for differences throughout. Participants were recruited through a non-probability web-panel and the survey was administered online, likely introducing a selection bias in favour of more digitally literate participants. This is important given that the primary outcome of interest (access to online testing) is likely to be affected by a person’s digital literacy. Experiences among those who find online access most challenging may not have been captured. The findings are therefore likely to be a conservative estimate of the true differences between those engaging online and not engaging online.

## Conclusions

This analysis shows a snapshot of STI testing access among sexual health service users during the first year of the COVID-19 pandemic. Within an increasingly digital healthcare landscape, the pandemic accelerated the roll-out of digital health solutions in sexual health services.^22^ Assessing who benefits from digital solutions and who may be excluded is crucial within this context. These data suggest that even in a pandemic context, where many people censored their health needs,^23^ the most at risk of adverse sexual health were less likely to access online care, potentially exacerbating existing inequalities. Ensuring that people with STI testing needs can easily and comfortably access their preferred method of testing should be prioritised by sexual healthcare providers.

Future work with a larger sample size of STI testers and online service users would allow a further exploration of the differences in access identified in this study. However, despite small numbers of online users in this study, there are signals that inequalities existed in regard to accessing services online. As this survey was administered online, future research reaching those unable or unwilling to engage online may reveal further gaps in access to online testing. Whether the existence of these inequalities is a COVID-specific finding or one that persists post-pandemic warrants further investigation as well. Further research is needed to know if those accessing online services less frequently simply prefer other types of services, or if they are facing barriers in accessing online STI testing. These differences in access patterns are particularly relevant as asymptomatic testing is increasingly offered online, and could cause inequalities in access to care if online testing is the only option offered to users. If differences in access patterns are found to be indicative of inequalities in access to care more broadly, mitigation strategies should be adopted by sexual health service providers.

## Supplemental Material

Supplemental Material - Who accessed STI testing in Britain during the COVID-19 pandemic and how: Findings from Natsal-COVID, a cross-sectional quasi-representative survey Supplemental Material for Who accessed STI testing in Britain during the COVID-19 pandemic and how: Findings from Natsal-COVID, a cross-sectional quasi-representative survey by Nuria Gallego Marquez, Alison R. Howarth, Emily Dema, Fiona Burns, Andrew J. Copas, Catherine H. Mercer, Pam Sonnenberg, Kirstin R. Mitchell, Nigel Field, Jo Gibbs in International Journal of STD & AIDS.

## Data Availability

The data used in this study are available in a public, open access repository: https://beta.ukdataservice.ac.uk/datacatalogue/studies/study?id=8865.
