# Supplementary material for: Who accessed STI testing in Britain during the COVID-19 pandemic and how: Findings from Natsal-COVID, a cross-sectional quasi-representative survey
Source: Int J STD AIDS. 2024 Sep 11;35(13):1055–71. doi: 10.1177/09564624241277582 (PMC11481401; doi:10.1177/09564624241277582)
Supplement: Supplemental Material - Who accessed STI testing in Britain during the COVID-19 pandemic and how: Findings from Natsal-COVID, a cross-sectional quasi-representative survey [file sj-pdf-1-std-10.1177_09564624241277582.pdf]

Appendix 1. Comparison of unweighted and weighted sample demographic profiles

	All				Men				Women			
	Unweighted		Weighted		Unweighted		Weighted		Unweighted		Weighted	
	n	%	n	% (95% CI)	n	%	n	% (95% CI)	n	%	n	% (95% CI)
**Age**												
18-24	793	13.1	750	12.4 (11.5-13.3)	371	13.1	413	13.6 (12.2-15.1)	417	13.0	332	11.0 (9.97-12.2)
25-29	912	15.0	821	13.5 (12.7-14.5)	363	12.8	377	12.4 (11.2-13.8)	546	17.0	441	14.6 (13.5-15.9)
30-34	680	11.2	649	10.7 (9.90-11.6)	244	8.59	287	9.48 (8.30-10.8)	433	13.5	359	11.9 (10.8-13.1)
35-44	1484	24.4	1532	25.3 (24.1-26.5)	712	25.1	818	27.0 (25.2-28.9)	767	23.9	708	23.5 (22.0-25.1)
45-59	2203	36.3	2312	38.1 (36.8-39.5)	1150	40.5	1138	37.5 (35.6-39.5)	1051	32.7	1171	38.9 (37.1-40.7)
**Gender**												
Men	2840	46.8	3033	50.0 (48.7-51.4)								
Women	3214	52.9	3011	49.7 (48.3-51.0)								
Identifies in another way	18	0.30	20	0.32 (0.20-0.51)								
**Ethnicity**												
White	5320	88.8	5200	87.1 (86.0-88.1)	2456	88.0	2584	86.6 (85.0-88.1)	2848	89.4	2598	87.5 (86.1-88.8)
Mixed/multiple ethnicities	139	2.32	93	1.56 (1.30-1.88)	60	2.15	45	1.49 (1.13-1.98)	78	2.45	48	1.60 (1.27-2.03)
Asian/Asian British	353	5.89	428	7.17 (6.44-7.97)	182	6.52	225	7.54 (6.48-8.77)	171	5.37	203	6.84 (5.87-7.94)
Black/African/Caribbean/ Black British	151	2.52	180	3.01 (2.54-3.57)	80	2.87	87	2.93 (2.32-3.69)	71	2.23	93	3.12 (2.44-3.99)
Other ethnic group	30	0.50	70	1.17 (0.78-1.76)	12	0.43	42	1.40 (0.77-2.52)	18	0.56	28	0.96 (0.57-1.59)
**Education**												
No qualification	299	4.92	313	5.15 (4.56-5.81)	152	5.35	171	5.65 (4.75-6.70)	147	4.57	141	4.69 (3.96-5.55)
Below degree	2913	48.0	3025	49.9 (48.5-51.3)	1358	47.8	1549	51.1 (49.0-53.1)	1548	48.2	1468	48.8 (46.9-50.6)
Degree	2860	47.1	2727	45.0 (43.6-46.3)	1330	46.8	1313	43.3 (41.3-45.3)	1519	47.3	1402	46.6 (44.7-48.4)
**Social grade**												
Upper middle class or middle class	1790	29.5	1399	23.1 (22.0-24.1)	1006	35.4	714	23.5 (22.1-25.1)	778	24.2	678	22.5 (21.1-24.0)
Lower middle class or skilled working class	2800	46.1	3219	53.1 (51.7-54.4)	1176	41.4	1620	53.4 (51.4-55.4)	1617	50.3	1591	52.8 (51.0-54.7)

Working class or lower level of subsistence	1482	24.4	1447	23.9 (22.7-25.0)	658	23.2	699	23.1 (21.4-24.8)	819	25.5	742	24.6 (23.1-26.2)
**Region**												
England	5312	87.5	5256	86.7 (85.7-87.6)	2492	87.8	2638	87.0 (85.5-88.3)	2807	87.3	2604	86.5 (85.1-87.7)
Wales	275	4.53	290	4.78 (4.21-5.41)	124	4.37	140	4.63 (3.82-5.59)	150	4.67	148	4.92 (4.17-5.80)
Scotland	485	7.99	519	8.55 (7.79-9.37)	224	7.89	255	8.40 (7.30-9.65)	257	8.00	259	8.61 (7.60-9.73)
**Sexuality**												
Heterosexual or straight	5337	88.7	5768	96.2 (95.8-96.5)	2472	87.9	2885	96.2 (95.7-96.7)	2863	89.8	2881	96.6 (96.2-97.0)
Gay or Lesbian	281	4.67	107	1.79 (1.56-2.04)	194	6.90	71	2.37 (2.03-2.76)	83	2.60	32	1.07 (0.83-1.37)
Bisexual	331	5.50	84	1.41 (1.23-1.61)	120	4.27	27	0.90 (0.70-1.14)	205	6.43	51	1.71 (1.47-1.98)
Other	67	1.11	40	0.66 (0.49-0.89)	25	0.89	15	0.50 (0.29-0.86)	36	1.13	18	0.60 (0.42-0.86)
**Same-sex partner in the last 5 years**												
No	5551	92.8	5758	96.3 (95.8-96.7)	2530	90.6	2848	95.4 (94.6-96.1)	3003	94.7	2891	97.2 (96.6-97.7)
Yes	432	7.22	222	3.71 (3.28-4.19)	263	9.42	139	4.64 (3.94-5.45)	169	5.33	83	2.79 (2.32-3.36)
**Relationship status**												
Married or in a steady relationship	4301	71.2	4306	71.5 (70.2-72.7)	1953	69.4	2081	69.2 (67.3-71.1)	2338	73.0	2215	73.8 (72.2-75.4)
In a new or casual relationship	279	4.62	293	4.86 (4.28-5.51)	135	4.79	163	5.44 (4.54-6.50)	142	4.43	127	4.24 (3.55-5.05)
Not currently in a relationship or at the end of a relationship (e.g., separating)	1401	23.2	1368	22.7 (21.6-23.9)	699	24.8	728	24.2 (22.5-26.0)	699	21.8	637	21.2 (19.8-22.8)
In more than one type of relationship	14	0.23	14	0.24 (0.12-0.45)	6	0.21	9	0.30 (0.12-0.75)	8	0.25	5	0.17 (0.01-0.39)
Other	43	0.71	44	0.73 (0.53-1.00)	23	0.82	24	0.80 (0.51-1.24)	18	0.56	18	0.58 (0.36-0.94)
**Cohabitation status**												
Married/in a steady relationship and cohabitating	3761	62.3	3796	63.0 (61.7-64.3)	1742	61.9	1853	61.7 (59.6-63.7)	2013	62.8	1936	64.5 (62.8-66.2)
Married/in a steady relationship and not cohabitating	540	8.94	510	8.47 (7.75-9.25)	211	7.49	228	7.57 (6.56-8.74)	325	10.1	278	9.28 (8.30-10.4)
Not in a steady relationship	1737	28.8	1719	28.5 (27.3-29.8)	863	30.7	924	30.8 (28.9-32.7)	867	27.1	787	26.2 (24.6-27.9)
**Employment status**												
Employed	4306	70.9	4331	71.4 (70.2-72.6)	2174	76.6	2336	77.0 (75.3-78.7)	2125	66.1	1987	66.0 (64.2-67.7)

Employed but on paid leave (including furlough)	330	5.43	314	5.18 (4.61-5.81)	130	4.58	136	4.48 (3.70-5.41)	198	6.16	176	5.85 (5.06-6.75)
Unemployed	643	10.6	652	10.8 (9.93-11.6)	325	11.4	351	11.6 (10.3-13.0)	314	9.77	297	9.85 (8.80-11.0)
Student	285	4.69	248	4.09 (3.60-4.65)	101	3.56	102	3.35 (2.70-4.14)	180	5.60	142	4.72 (4.02-5.54)
Other (incl retired, homemaker, etc.)	508	8.37	519	8.55 (7.83-9.33)	110	3.87	108	3.57 (2.92-4.37)	397	12.4	409	13.6 (12.4-14.9)
**Became unemployed since the first lockdown**												
No	5564	93.1	5574	93.4 (92.7-94.1)	2609	93.6	2787	93.6 (92.5-94.6)	2940	92.7	2771	93.2 (92.3-94.1)
Yes	414	6.93	393	6.59 (5.94-7.31)	179	6.42	190	6.39 (5.44-7.49)	233	7.34	201	6.77 (5.91-7.74)
**Furloughed under Coronavirus Job Retention Scheme**												
No	5086	85.1	5084	85.2 (84.2-86.2)	2368	84.9	2522	84.7 (83.1-86.2)	2705	85.3	2548	85.7 (84.4-87.0)
Yes	892	14.9	884	14.8 (13.9-15.8)	420	15.1	455	15.3 (13.8-16.9)	468	14.8	424	14.3 (13.1-15.6)
**Currently smokes cigarettes**												
No	4629	76.7	4577	75.9 (74.7-77.1)	2066	73.3	2161	71.7 (69.8-73.6)	2553	79.8	2406	81.3 (78.7-81.7)
Yes	1406	23.3	1452	24.1 (22.9-25.3)	753	26.7	851	28.3 (26.4-30.2)	645	20.2	592	19.8 (18.3-21.3)
**Number of days drinking in the last week**												
0 days	2318	38.3	2307	38.2 (36.8-39.5)	910	32.1	964	31.9 (30.0-33.8)	1400	43.7	1334	44.4 (42.6-46.3)
1-2 days	2217	36.6	2249	37.2 (35.9-38.5)	1043	36.8	1166	38.5 (36.5-40.6)	1168	36.4	1077	35.9 (34.1-37.6)
3-4 days	940	15.5	926	15.3 (14.4-16.3)	531	18.7	544	18.0 (16.5-19.6)	405	12.6	378	12.6 (11.4-13.8)
5-7 days	582	9.61	565	9.35 (8.58-10.2)	349	12.3	351	11.6 (10.4-12.9)	233	7.27	215	7.15 (6.26-8.14)
**Depression (PHQ2 score)**												
No symptoms of depression (0-2)	4030	67.5	4046	67.8 (66.5-69.1)	1905	68.2	2010	67.3 (65.4-69.3)	2119	67.0	2029	68.4 (66.7-70.1)
Symptoms of depression (3-6)	1943	32.5	1923	32.2 (30.9-33.5)	888	31.8	975	32.7 (30.7-34.7)	1044	33.0	936	31.6 (29.9-33.3)
**Anxiety (GAD2 score)**												
No symptoms of anxiety (0-2)	4057	67.5	4119	68.6 (67.3-69.9)	2012	71.8	2129	71.1 (69.1-72.9)	2042	64.0	1986	66.5 (64.8-68.2)
Symptoms of anxiety (3-6)	1953	32.5	1882	31.4 (30.1-32.7)	792	28.3	868	29.0 (27.1-30.9)	1148	36.0	1000	33.5 (31.8-35.2)
**General health**												
Bad/very bad	406	6.71	391	6.46 (5.83-7.17)	174	6.15	177	5.83 (4.96-6.85)	229	7.14	211	7.03 (6.13-8.04)
Fair	1433	23.7	1437	23.8 (22.6-25.0)	665	23.5	728	24.1 (22.4-25.9)	760	23.7	700	23.3 (21.8-24.9)
Good/very good	4216	69.6	4221	69.8 (68.5-71.0)	1991	70.4	2120	70.1 (68.2-71.9)	2219	69.2	2095	69.7 (68.0-71.4)

**Disability**												
None	3885	65.1	3959	66.4 (65.1-67.7)	1917	68.8	2077	69.8 (67.8-71.7)	1966	62.1	1880	63.4 (61.6-65.1)
Yes, not limiting	550	9.21	515	8.63 (7.91-9.42)	233	8.36	224	7.51 (6.53-8.62)	317	10.0	291	9.82 (8.78-11.0)
Yes, limiting	1534	25.7	1488	25.0 (23.8-26.2)	638	22.9	676	22.7 (21.0-24.5)	882	27.9	796	26.8 (25.2-28.5)
